# Dose Response of MARV/Angola Infection in Cynomolgus Macaques following IM or Aerosol Exposure

**DOI:** 10.1371/journal.pone.0138843

**Published:** 2015-09-28

**Authors:** Sara C. Johnston, Kenny L. Lin, Nancy A. Twenhafel, Jo Lynne W. Raymond, Joshua D. Shamblin, Suzanne E. Wollen, Carly B. Wlazlowski, Eric R. Wilkinson, Miriam A. Botto, Arthur J. Goff

**Affiliations:** 1 Virology Division, United States Army Medical Research Institute of Infectious Diseases, Fort Detrick, Maryland, 21702, United States of America; 2 Nonclinical Development Division, United States Army Medical Research Institute of Infectious Diseases, Fort Detrick, Maryland, 21702, United States of America; 3 Pathology Division, United States Army Medical Research Institute of Infectious Diseases, Fort Detrick, Maryland, 21702, United States of America; Division of Clinical Research, UNITED STATES

## Abstract

Marburg virus infection in humans causes a hemorrhagic disease with a high case fatality rate. Countermeasure development requires the use of well-characterized animal models that mimic human disease. To further characterize the cynomolgus macaque model of MARV/Angola, two independent dose response studies were performed using the intramuscular or aerosol routes of exposure. All animals succumbed at the lowest target dose; therefore, a dose effect could not be determined. For intramuscular-exposed animals, 100 PFU was the first target dose that was not significantly different than higher target doses in terms of time to disposition, clinical pathology, and histopathology. Although a significant difference was not observed between aerosol-exposed animals in the 10 PFU and 100 PFU target dose groups, 100 PFU was determined to be the lowest target dose that could be consistently obtained and accurately titrated in aerosol studies.

## Introduction

Viruses of the family *Filoviridae* are single-stranded, negative-sense RNA, filamentous viruses that cause severe and often lethal hemorrhagic fever in humans and non-human primates (NHP). The family *Filoviridae* includes three genera: *Marburgvirus*, *Ebolavirus*, and *Cuevavirus*. The *Ebolavirus* genus consists of five distinct species: *Taï Forest ebolavirus*, *Reston ebolavirus*, *Sudan ebolavirus*, *Zaire ebolavirus*, and *Bundibugyo ebolavirus* [1]. The only species belonging to the *Marburgvirus* genus, *Marburg marburgvirus* (formerly *Lake Victoria marburgvirus*) has 2 distinct members: Marburg virus (MARV) and Ravn virus. Since its identification in Germany (former West Germany) and Serbia (former Yugoslavia) in 1967, sporadic outbreaks of MARV disease have occurred in Africa [2–6]. MARV has historically had approximately 50% case-fatality rate in humans [7]; however, large outbreaks in the Democratic Republic of the Congo and Angola have had case-fatality rates greater than 80% [7]. Transmission occurs through contact with critically ill (or recently deceased) patients or animals, or following contact with contaminated tissues or blood [8, 9].

The average incubation period for MARV infection in humans is 5–9 days, and this is followed by an approximately 5 day febrile phase characterized by influenza-like symptoms [3, 10] and the development of a maculopapular rash [11]. At 5–13 days following the onset of disease, the patient enters an “early organ” phase characterized by pyrexia, dyspnea, increased vascular permeability, and neurologic manifestations (delirium, irritability, and encephalitis) [3, 12–14]. Increased vascular permeability and subsequent hemorrhage result in the appearance of petechiae, mucosal bleeding, bleeding from sites of venipuncture, visceral hemorrhagic effusions, melena, bloody diarrhea, hematemesis, and ecchymoses [3, 12–14]. Critical disease develops as the patient progresses into the “late organ” phase, beginning around 2 weeks after symptom onset, characterized by cytolysis, widespread inflammation, and hemorrhage associated with disseminated intravascular coagulation (DIC); shock, multiorgan failure, and death may occur [3, 12, 13, 15].

Although MARV is often associated with limited outbreaks in remote regions of Africa, the high case fatality rate and low infectious dose make MARV a significant concern from a biodefense perspective. Marburgviruses have been classified as Category A agents by the United States Centers for Disease Control and Prevention, and there are currently no approved countermeasures for use against these or other filoviruses. Filovirus countermeasure development requires the use of well-characterized animal models that exhibit disease signs and symptoms similar to those seen in human disease cases. Understanding of these animal models begins with a determination of the appropriate amount of virus required to elicit disease and mortality through the use of dose response studies.

To date, the dose response for MARV/Angola has not been empirically demonstrated with statistical power. A 2010 manuscript published by Alves *et al* [16] detailed aerosol infection of cynomolgus macaques with MARV/Angola. In this study, 6 animals were divided into 2 dose groups: a low dose group and a high dose group. The low dose group received 2, 11, or 14 plaque forming units (PFU) of MARV (mean = 9 PFU); the high dose group received 99, 339, or 705 PFU of MARV (mean = 381 PFU). All animals succumbed to disease on days 7–9, with a mean time to disposition of 8.33 days. However, the study was not powered to assess the differences between dose groups. In the current study, the results of two statistically powered dose response studies for MARV/Angola in cynomolgus macaques are described; one following intramuscular (IM) exposure and the other following aerosol exposure. All animals succumbed to disease; differences in time to disposition, clinical pathology, and histopathology were not observed at doses ≥ 100 PFU. The data demonstrate that the dose of MARV/Angola that results in 50% lethality in cynomolgus macaques is less than 1 PFU following IM exposure and less than 10 PFU following aerosol exposure.

## Results

### Lethality of MARV/Angola in Cynomolgus Macaques

**Fig 1:**
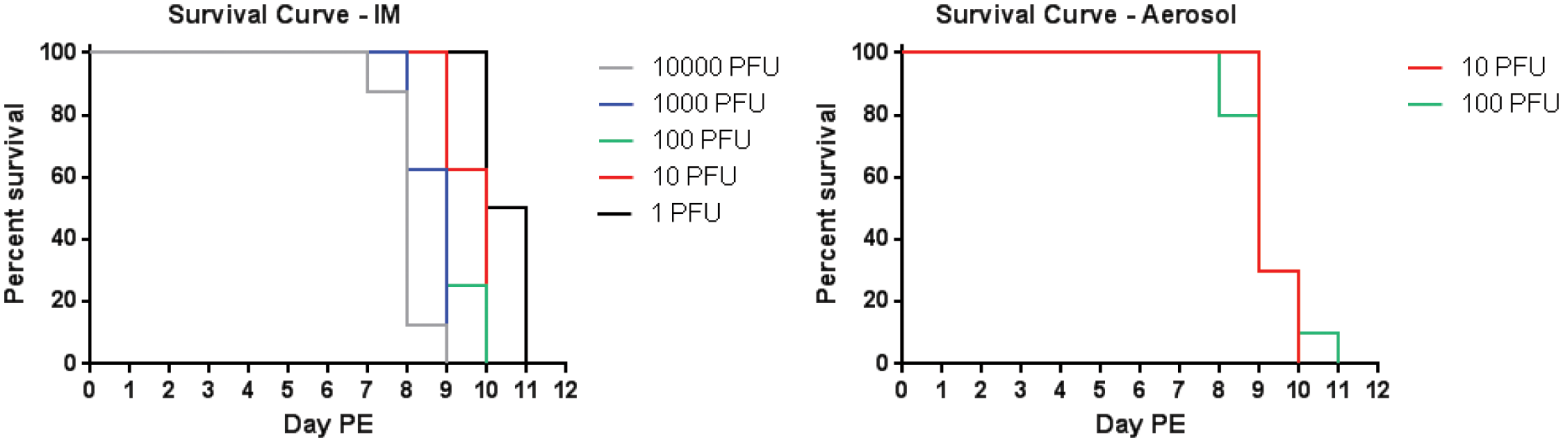
Kaplan-Meier survival curves. Animals were challenged by either the IM (A) or aerosol (B) routes with various concentrations of MARV/Angola (dose groups, in PFU, of virus are shown in the panel to the right of each graph). Shown are Kaplan-Meier curves depicting the percent survival for each dose group.

Two independent studies were conducted to assess the dose response of cynomolgus macaques to MARV/Angola following aerosol or IM exposure. In the first study, 40 animals randomized into 5 dose groups containing 8 animals each were exposed to MARV/Angola by the IM route. The target doses for the groups were 10,000 PFU, 1,000 PFU, 100 PFU, 10 PFU, and 1 PFU. The breakdown of the groups and the actual doses received (as determined by plaque assay) can be found in Table 1. All animals in each dose group succumbed to disease by day 11 post exposure (PE) (Fig 1). Pairwise statistical comparisons of the dose groups indicated that a significant difference existed between the 1 PFU group and all groups at or above 100 PFU in regards to time to disposition. There was also a significant difference between the 10 PFU group and the 10,000 PFU group. The 100 PFU dose group was the first dose group which was not significantly different than any higher dose group in regards to time to disposition.

**Table 1:** Exposure doses for cynomolgus macaques exposed to MARV/Angola by the IM or aerosol route.

Exposure Route	Target Dose (PFU)	Actual Dose (PFU)	Dose Range (PFU)
IM	1	0	N/A
IM	10	18.3	N/A
IM	100	260.0	N/A
IM	1,000	2,520.0	N/A
IM	10,000	29,200.0	N/A
Aerosol	10	33.1	8.2–59.3
Aerosol	100	235.4	149.9–365.5

100% mortality was observed in the IM study; therefore, a step-wise approach was taken for the aerosol study. In iteration one, twenty animals randomized into 2 dose groups containing 10 animals each were exposed to MARV/Angola by the aerosol route. The target doses for the groups were 100 PFU and 10 PFU. The makeup of the groups and the actual doses received (as determined by plaque assay) can be found in Table 1. If less than 100% mortality was seen with iteration one, then a second iteration would have been done at the higher doses. However, all animals in each dose group succumbed to disease by 11 days PE (Fig 1); therefore, a second iteration was not performed. The log-rank test for homogeneity of Kaplan-Meier in this study had *p* = 0.3537; therefore, this test provides no evidence of a significant difference in survival between dose groups.

### Clinical Observations

**Fig 2:**
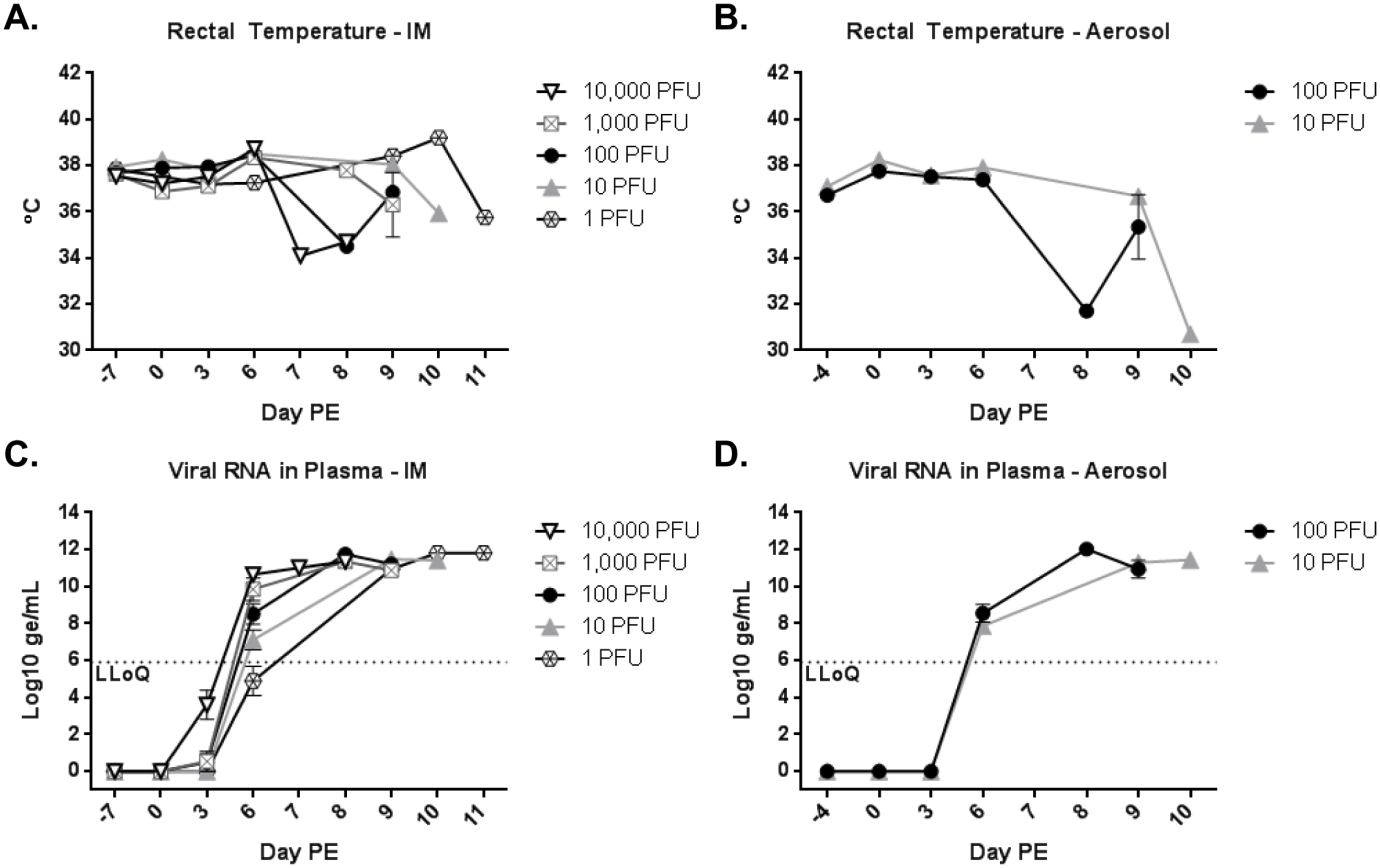
Rectal temperatures and viral RNA in blood. Rectal temperatures were measured on all blood collection days and prior to euthanasia. (A) shows rectal temperatures by dose group for IM-exposed animals, and (B) shows rectal temperatures by dose group for the aerosol-exposed animals. Viral RNA in the blood was measured by MARV-specific qRT-PCR and is reported in Log_10_ ge/mL for each dose group. (A) is the IM route and (B) is the aerosol route.

Daily observations were performed on all animals and physical examinations under anesthesia were performed prior to all blood collections. Additional observations were performed after the first signs of illness. Onset of initial signs occurred between 7–9 days PE for aerosol-exposed animals, and between 6–9 days PE for IM-exposed animals. These included macular rash, weakness, staggering, lymphadenopathy, bleeding, facial edema, anorexia, changes in rectal temperatures, and labored breathing (aerosol-exposed animals only). Disease progression was rapid, and most animals became moribund within 24–48 hours of initial symptom onset. Sharp drops in rectal temperatures were observed for moribund animals (Fig 2).

The amount of viral RNA present in plasma was determined using a MARV/Angola qRT-PCR assay that has been validated at USAMRIID. EDTA plasma samples for PCR were collected on scheduled blood collection days and before euthanasia when animals were observed to be moribund. MARV genomes in the plasma were detected above the lower limit of quantification for the assay by 6 days PE (Fig 2). The only exception to this was the IM-exposed 1 PFU group for which RNA levels did not exceed the lower limit of quantification until 9 days PE. For IM-exposed animals, the level of detection 6 days PE correlated well with the dose of virus received; however, any stratification by dose disappeared by 9 days PE as RNA levels reached 10–12 Log_10_ genome equivalents (ge)/mL (Fig 2). Dose stratifications were not observed for aerosol-exposed animals. Similar to IM-exposed animals, peak levels of 10–12 Log_10_ ge/mL were observed 8–9 days PE (Fig 2).

### Clinical Pathology

**Fig 3:**
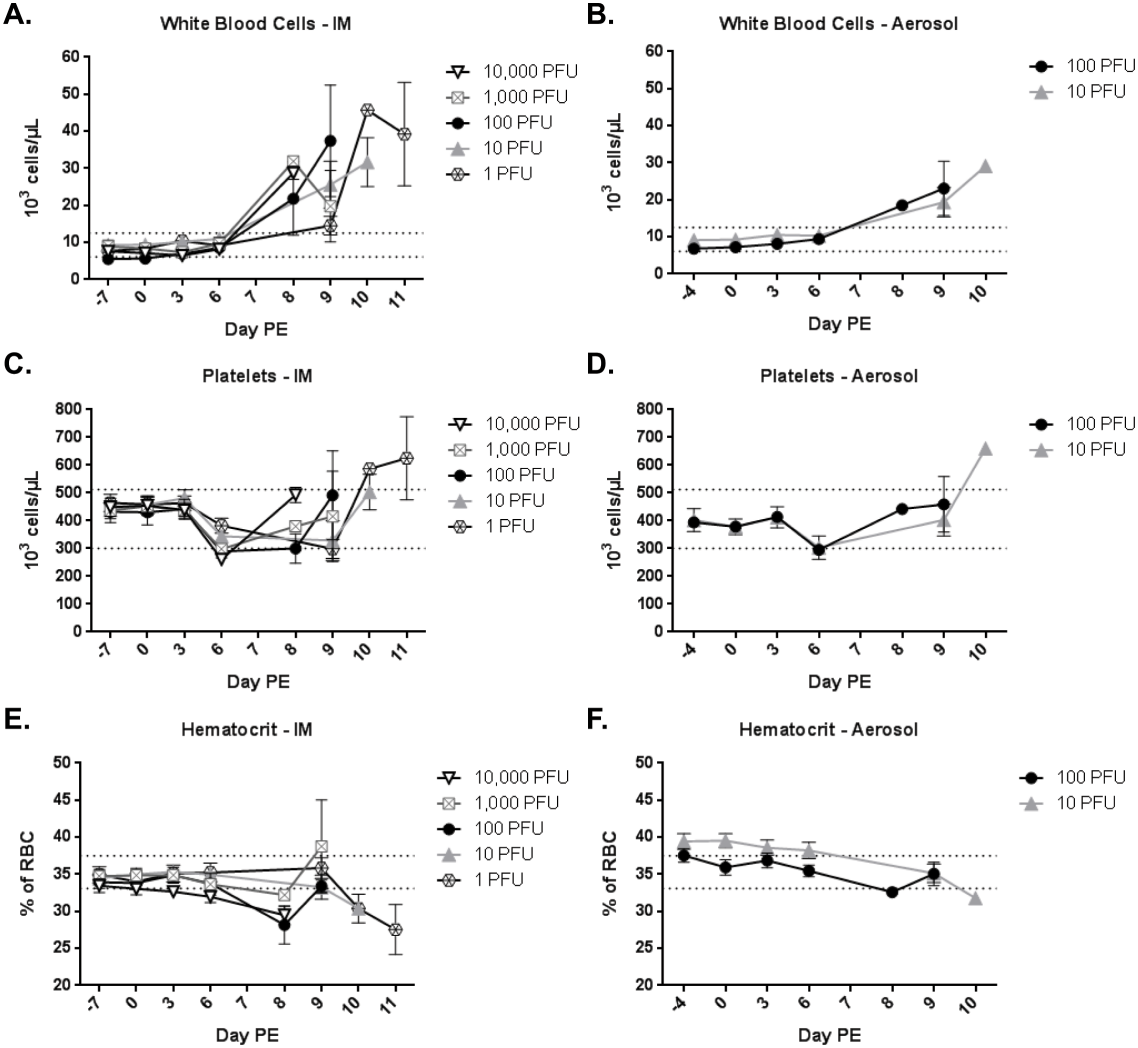
Hematology. Hematology was performed following each blood collection. (A), (C), and (E) show hematology parameters for IM-exposed dose groups. (B), (D), and (F) show hematology parameters for aerosol-exposed dose groups.

**Fig 4:**
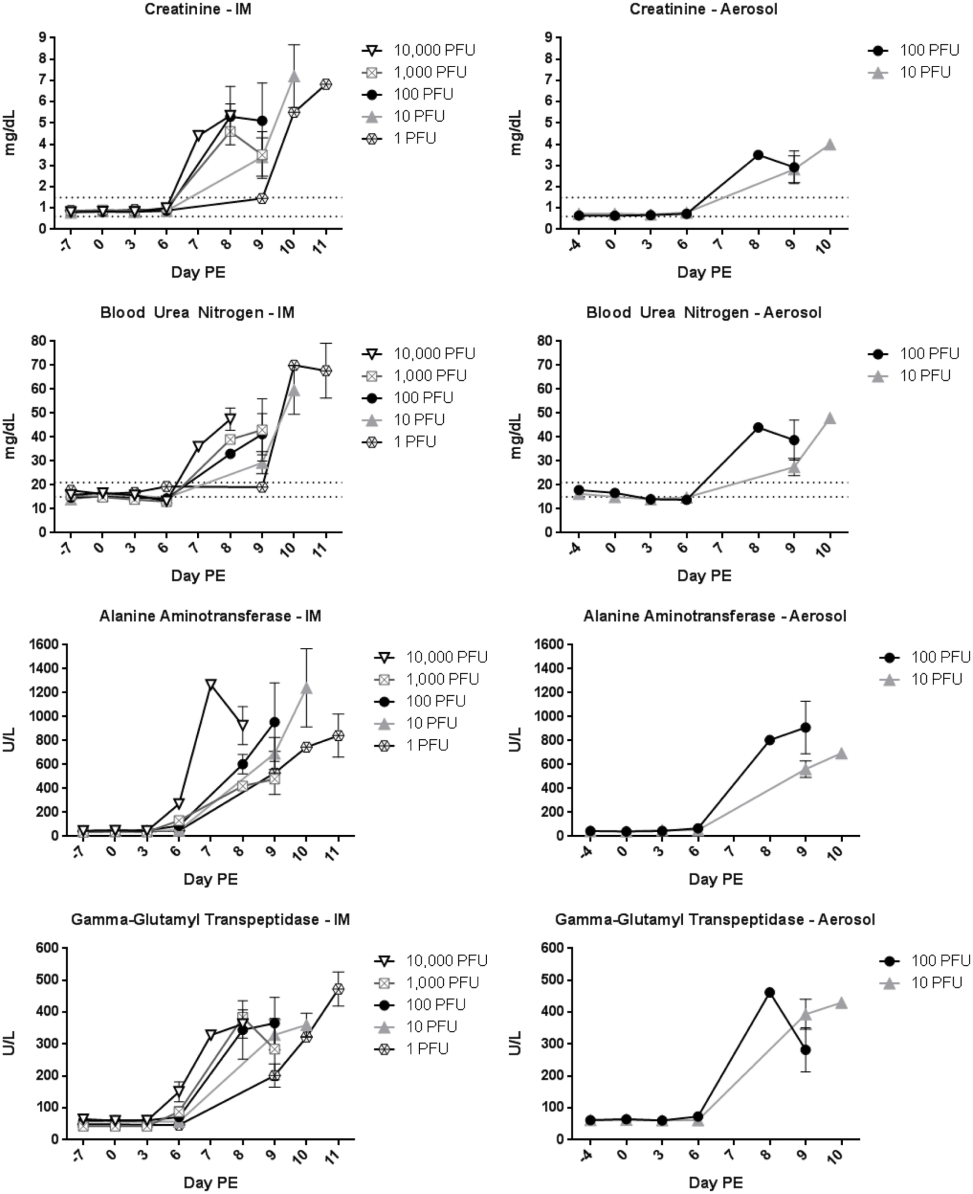
Clinical chemistries. Clinical chemistry measurements were obtained following each blood collection. (A), (C), (E), and (G) show chemistry parameters for IM-exposed dose groups. (B), (D), (F), and (H) show chemistry parameters for aerosol-exposed dose groups.

EDTA whole blood was collected for hematology analyses and serum was collected for clinical chemistry analyses. For IM-exposed animals, the graphs contain data for only one animal on day 7 PE for the 10,000 PFU group, one animal on day 8 PE for the 1,000 PFU group, and one animal on day 10 PE for the 1 PFU group. For aerosol-exposed animals, the graphs contain data for only one animal on day 8 PE for the 100 PFU group and one animal on day 10 PE for the 10 PFU group. All other data points contain ≥ 2 animals. General trends were similar for animals challenged by the IM and aerosol routes. Increases in total white blood cells (WBC) were observed by 8–9 days PE (Fig 3). A slight drop in lymphocyte numbers was seen on day 6 PE (S1 Fig), and sharp increases in lymphocytes corresponding to increases in WBC were noted as animals became moribund. This is most likely attributed to splenic contraction. A slight reduction in platelet levels was also noted on day 6 PE, but numbers were actually elevated above baseline at the time of disposition (Fig 3). Reductions in hematocrit (red blood cell count and hemoglobin), were minimal for most dose groups (Fig 3 and S1 Fig); however, in addition to splenic contraction, dehydration and hemoconcentration may artificially elevate the HCT. The most noticeable reductions occurred for animals that succumbed days 10–11 PE likely due to hemorrhage. Blood urea nitrogen and creatinine were elevated on the day of disposition for most animals (Fig 4), suggesting the animals were azotemic. The liver enzymes alanine aminotransferase, aspartate aminotransferase, alkaline phosphatase, and gamma-glutamyl transpeptidase were all extremely elevated by 8–9 days PE caused by acute severe liver disease (Fig 4 and S2 Fig). Albumin and total protein were reduced (S2 Fig) and, similar to hematocrit, dehydration may artificially elevate albumin and total protein levels. Declining levels of albumin and total proteins (such as globulins) indicate decreased liver function (inability to produce these proteins) as well as loss through hemorrhage.

Statistical analyses comparing IM dose groups revealed differences in clinical pathology measurements between the lowest two dose groups (target dose of 1 PFU or 10 PFU) and higher groups (target dose of 100 PFU, 1,000 PFU, or 10,000 PFU); however, that significance was dramatically reduced once the 100 PFU group was compared to the 1,000 PFU and 10,000 PFU group. Similar statistical analyses did not reveal dose stratifications for aerosol challenged animals.

### Histopathology and Immunohistochemistry

The most common histological changes for aerosol and IM challenged animals included necrotizing lymphadenitis of the tracheobronchial (aerosol) and inguinal (IM) lymph nodes; lymphocytolysis with lymphoid depletion in many lymph tissues; widespread degeneration and necrosis of hepatocytes with few intracytoplasmic inclusion bodies; necrotizing splenitis; pulmonary congestion alveolar histiocytosis (aerosol); interstitial pneumonia; edema, fibrin, alveolar histiocytosis (aerosol), multisystemic vasculitis; thromboemboli; fibrin deposition; and hemorrhage. The severity of histologic changes in all animals was consistent in all dose groups.

Immunohistochemistry (IHC) was used to identify cells with MARV/Angola antigen in representative sections of the liver, spleen, tracheobronchial, and inguinal lymph nodes. The intensity of the staining in all animals was consistent regardless of challenge route or challenge dose and supported systemic infection with MARV/Angola in all animals. Of the 3 tissues, the spleen and liver were the most intensely immunopositive.

## Discussion

The development of well-characterized nonhuman primate models is necessary for the testing and evaluation of filovirus countermeasures. Aside from understanding virus pathogenesis, the appropriate amount of virus required to elicit morbidity and mortality must be empirically determined. To date, the dose response for MARV/Angola has not been demonstrated with statistical power. Here, we describe the results of two statistically-weighted studies aimed at defining the dose response for MARV/Angola in cynomolgus macaques following IM or aerosol exposure. The clinical characteristics of MARV/Angola in cynomolgus macaques following aerosol or IM exposure were similar to those seen in humans; rash, coagulopathy, azotemia, and shock are prominent findings, and numerous tissues are affected. In addition, the data correlate well with published NHP studies in which MARV/Angola was used [16–18]. Since all animals in these studies succumbed, a true dose response cannot be determined.

Statistical analyses revealed significant differences in time to disposition and clinical pathology measurements for IM exposed animals between the 1 PFU group and all groups at or above 100 PFU; there was also a significant difference between the 10 PFU group and the 10,000 PFU group. Therefore, the 100 PFU dose group was the first IM exposed dose group which was not significantly different than any higher dose group. Dose stratifications were not noted for aerosol exposed animals.

The dose response studies for MARV/Angola described in this report were designed with the appropriate statistical power to demonstrate differences in survival, clinical pathology, and histopathology. For IM exposed animals, the 100 PFU, 1,000 PFU, and 10,000 PFU groups were indistinguishable. Although any of these doses could be justified for use in countermeasure studies, 10,000 PFU could potentially overwhelm the system and/or elicit a vaccine effect. This could result in either failure and subsequent exclusion of countermeasures that might be effective in an appropriately balanced system or inclusion of countermeasures that, due to a vaccine effect caused by the large concentration of virus, may only be minimally effective when tested in an appropriately balanced system. For aerosol exposed animals, the 10 PFU and 100 PFU groups were indistinguishable. Again, either of these doses could be justified for countermeasure development; however, 10 PFU is the lower limit of what can accurately be measured and administered by aerosol systems and thus is a more difficult dose to consistently deliver within a half log acceptable range of the target dose. Although we did not challenge with 1,000 PFU of MARV/Angola by aerosol in this study, clinical characteristics of the 100 PFU group were similar to those described in the literature for animals infected with 705 PFU of MARV/Angola [16]. Therefore, the data suggest that animals exposed to 100–1000 PFU of MARV/Angola by the aerosol or IM route display consistent disease symptoms that are reproducible and accurately depict human disease. However, the dose of MARV/Angola ultimately chosen for countermeasure studies should be study dependent and based on the ultimate goal of the study and an empirical evaluation of the system to be used.

## Materials and Methods

### Ethics Statement

Research was conducted under an animal protocol approved by the IACUC at the United States Army Medical Research Institute of Infectious Diseases (USAMRIID). This protocol covered all of the experiments performed during this study, including the method of euthanasia, and complied with the Animal Welfare Act, PHS Policy, and other Federal statutes and regulations relating to animals and experiments involving animals. The facility where this research was conducted is accredited by the Association for Assessment and Accreditation of Laboratory Animal Care, International and adheres to principles stated in the Guide for the Care and Use of Laboratory Animals, National Research Council, 2011. Animals were housed one per cage in stainless steel cages with squeeze capabilities for handling in ABSL-4 biocontainment, and appropriate enrichment (i.e. toys, mirrors, etc.) was provided. Animals were acclimated in BSL-4 animal rooms for 3–7 days prior to study initiation. During the in-life portion of the study, animals were provided 2050C Teklad Monkey Chow (Harlan Laboratories, Indianapolis, IN) and water *ad libitum* via an automatic watering system. Oral rehydration solution (Pedialyte®) was provided, immediately following challenge and throughout the in-life phase, in a bottle attached to the front of the cage in a manner that an animal could drink, even if lying down. Additionally, the animals were provided hydrating fruits, liquids, and other food items, as determined appropriate by the investigators or USAMRIID Veterinary Medicine Division staff. Animals were anesthetized prior to handling (such as for physical examinations and blood collections) and were euthanized when moribund or at the end of study after deep anesthesia by intracardiac administration of a pentobarbital-based euthanasia solution.

To alleviate suffering, moribund animals were euthanized under deep anesthesia. To be declared moribund, an animal met the following criteria:

- Unresponsive; or
- Moderate to dramatically reduced response to external stimuli and rectal temperature ≤ 34°C; or
- Moderate to dramatically reduced response to external stimuli and 2 or more of the following:
  Blood urea nitrogen ≥ 68 mg/dL
  Creatinine ≥ 2.8 mg/dL
  Gamma-glutamyl transpeptidase ≥ 391 U/dL
  Calcium ≤ 6.8 mg/dL
  Glucose ≤ 47 mg/dl (applicable only when performed within 1 hour of collection)

### Animals and Group Size Justification

Two separate studies were conducted, to be referred to as the IM study and the aerosol study. In the IM study, 40 research-naive adult cynomolgus macaques (*Macaca fascicularis*), mixed male and female, of Asian origin were obtained from Worldwide Primates (Miami, FL) and randomized based on weight, age, and sex by the study statistician into 5 groups of 8 animals. The target doses for the 5 groups were 1 PFU, 10 PFU, 100 PFU, 1,000 PFU, and 10,000 PFU. In the aerosol study, 20 research-naive adult cynomolgus macaques (*Macaca fascicularis*), mixed male and female, of Asian origin were obtained from Primate Products (Immokalee, FL) and randomized based on weight, age, and sex by the study statistician into 2 groups of 10 animals. Numerical simulations using SAS Probit indicated that a minimum of 4 groups spanning survival rates of 0% to 100% with 8–10 animals per group yield stable estimates of the dose response with confidence intervals reaching out to 0.3 log10 (approximately 2.0-fold) under monotone assumptions of response profiles for intermediate doses. When sample size is 8–10 animals per group, the range of the 95% confidence interval of the log10 dose response is no larger than the increments between doses on a log10 scale.

Animals for both studies were prescreened and determined to be negative for filovirus (Marburg/Ebola), Herpes B, STLV-1, SIV, SRV1, 2, and 3 antibodies and TB, *Salmonella*, *Campylobacter*, hypermucoid HVM *Klebsiella*, and *Shigella* infections. In addition, all animals had passed a semi-annual physical examination and were certified as healthy by the attending veterinarian.

### Virus

Marburg virus (MARV) Angola variant [CDC #200501379 Angola 810820 (CDC SPB 810838)] was isolated from a fatal case in an eight month old female patient in Angola in 2005 (World Health Organization, 2005. Marburg haemorrhagic fever, Angola. Wkly. Epidemiol. Rec. 80:158–159). A 1:10 dilution was used to grow the master seed stock used on this study. The seed stock was analyzed for sterility, purity, nucleotide sequence, morphology, and confirmation of virulence. The seed stock had a particle count of 5.2 × 10^10^ particles and a titer of 3.5 × 10^7^ PFU/mL. On the day of challenge, day 0 PE, a stock of MARV was prepared by making the required dilutions in MEM Alpha GlutaMax-I (Life Technologies, Grand Island, NY) containing either 2% heat-inactivated fetal calf serum (Life Technologies, Grand Island, NY) (for IM exposures) or 10% heat-inactivated fetal calf serum (for aerosol exposures).

### Virus Exposures

In the IM study, animals were challenged IM in the right lateral thigh with 1.0 mL of MARV challenge material prepared to obtain the target dose of either 1, 10, 100, 1,000, or 10,000 PFU. A sample of the challenge material was agarose plaque-titrated to determine the injected PFU. In the aerosol study, animals were exposed to MARV challenge material prepared to obtain the target dose of either 10 or 100 PFU in the USAMRIID Head-Only Automated Bioaerosol Exposure System (ABES-II) System. The total volume of aerosol breathed was determined by the exposure time required to deliver the estimated inhaled dose (required exposure time was based on each animal’s minute volume). The aerosol challenge was generated using a Collison nebulizer to produce a highly respirable aerosol (flow rate 7.5 ± 0.2 L/minute). The system generated a target aerosol of 1–3 μm mass median aerodynamic diameter determined by TSI Aerodynamic Particle Sizer (TSI, Shoreview, MN). Samples of the pre-spray suspension (challenge material) and aerosol collected from the exposure chamber using an all glass impinger (AGI) during each challenge were agarose plaque-titrated to determine PFU. The aerosol challenge dose for each animal was calculated from AGI plaque-titration data using the minute volume determined with a plexiglass whole body plethysmograph box using Buxco XA software (Buxco Research Systems, Wilmington, NC).

### Animal Observations and Euthanasia

Animals were evaluated daily by study personnel. In the morning, animals were evaluated cage side for signs of illness (responsiveness, cough, edema, rash, bleeding, and motor function). Other observations such as biscuit/fruit consumption, condition of stool, and urine output were also documented, if possible. Animals were observed several times a day when clinical signs of illness were apparent. All observations occurred ≥ 4 hours apart. Euthanasia under deep anesthesia (> 6 mg/kg Telazol IM) by intracardiac administration of a pentobarbital-based euthanasia solution (0.3–0.4 ml/kg) was performed on all moribund animals.

Physical examinations (including rectal temperature measurements and blood collections) under anesthesia occurred on day -7/-4 and days 0, 3, 6, 9, and 12 PE. In addition, moribund animals anesthetized for euthanasia underwent a physical examination prior to the administration of the pentobarbital-based euthanasia solution.

### Clinical Pathology

For serum chemistries, whole blood was collected into Z Serum Clot Activator Greiner Vacuette tubes (Greiner Bio-One, Monroe, NC). Tubes were gently inverted by hand 5–10 times and placed upright. Tubes were allowed to clot for 30–60 minutes and the serum separated in a centrifuge set at 1800 x g for 10 minutes at ambient temperature. The required volume of serum was removed for chemistry analysis using a Piccolo Point-Of-Care Analyzer (Abaxis, Union City, CA). Serum was removed from the clot within 1 hour of centrifugation and was analyzed within 40–70 minutes of collection.

For hematology, whole blood was collected into Greiner Vacuette blood tubes containing the anti-coagulant K3 EDTA. Tubes were gently inverted by hand 5–10 times to ensure adequate mixing. Hematology was performed on the ADVIA 120 (Siemens Healthcare, Malvern, PA) for the aerosol study and the Hemavet 950 FS (Drew Scientific, Waterbury, CT) for the IM study. Following completion of the CBC analysis, the remaining blood was centrifuged to separate the plasma in a centrifuge set at 2500 x g for 10 minutes at ambient temperature. Centrifugation occurred within 6 hours of the addition of the blood to the EDTA tube. A 200 μl volume of EDTA plasma was added to 600 μl of TRIzol LS (Life Technologies, Grand Island, NY) in preparation for qRT-PCR.

### Extraction and qRT-PCR

Inactivated samples were extracted and eluted with AVE Buffer (Qiagen, Valencia, CA) using a QIAamp Viral RNA Mini Kit (Qiagen, Valencia, CA). All samples were used in qRT-PCR on an Applied Biosystems 7500 Fast Dx Real-Time PCR instrument (Life Technologies, Grand Island, NY). The RT-PCR reaction used the SuperScript II One-Step RT-PCR System (Life Technologies, Grand Island, NY) with additional MgSO_4_ added to a final concentration of 3.0 mM. The sequence of the primer and probes for the MARV/Angola glycoprotein (GP) gene are described below. The genomic equivalents were determined using a standard curve of synthetic RNA of known concentration.

Forward primer (0.6 μM): 5′—CCA GTT CCA GCA ATT ACA ATA CAT ACA—3′
Reverse primer (0.6 μM): 5′—GCA CCG TGG TCA GCA TAA GGA—3′
Probe (0.2 μM): 6FAM—CAA TAC CTT AAC CCC C—MGBNFQ

### Plaque Assay

A 1:10 dilution series ranging from 10^−1^ to 10^−6^ was prepared in 1X Plaque Assay Media [1X MEM (Corning Cellgro, Manassas, VA) containing 5% heat-inactivated fetal calf serum (Life Technologies, Grand Island, NY) and 1X Penicillin/Streptomycin (Corning Cellgro, Manassas, VA)] for each sample for plaque titration. In addition, a positive control sample consisting of MARV/Angola at a known concentration was similarly diluted in Plaque Assay Media. Media was removed by decanting from 6-well plates of Vero-E6 cells (BEI Resources, Manassas, VA), and 100–200 μL of each dilution was plated in triplicate. The plates were rocked gently by hand to ensure the cells were completely covered in inoculum and were placed in an incubator at 37 ± 2°C and 5% CO_2_ for 60 ± 10 min, with gentle rocking by hand every 15 ± 5 min. Melted 1% agarose was diluted 1:1 in 2X EBME Complete [2X EBME + HEPES (USAMRIID)] containing 10% heat-inactivated fetal calf serum (Life Technologies, Grand Island, NY) to create the primary overlay. To each well, 2 mL of primary overlay was added, and the plates were gently rocked and then allowed to solidify at ambient temperature. The plates were incubated at 37 ± 2°C and 5% CO_2_ for 7 days. A secondary overlay was created by making a primary overlay containing 4% Neutral Red (Life Technologies, Grand Island, NY), and 2 mL of secondary overlay was added to each well. The plates were gently rocked and then allowed to solidify at ambient temperature. The plates were incubated for 24 hr at 37 ± 2°C and 5% CO_2_, and virus plaques were subsequently counted. The mean plaque count for each dilution was multiplied by the dilution factor and divided by the volume of inoculum used (in mL) to derive the titer in PFU/mL. An assay was considered acceptable if the cells were 85–100% confluent at the start of the experiment, the negative control plate (Plaque Assay Media only) had no plaques, and the PFU/mL determined for the positive control sample was within 0.5 log of the known PFU/mL value for that sample.

### Necropsy

Necropsies were conducted at BSL-4 by a veterinary pathologist, or their designee, on all animals in this study. The following tissues were collected for histopathology on the animals: haired skin with tattoo, inguinal lymph node, tracheobronchial lymph node, spleen, liver, and lungs. Tissues were fixed in neutral buffered formalin for a minimum of 21 days prior to removal from BSL-4 and processing for histology and immunohistochemistry (IHC).

### Histology

The tissue samples were trimmed, routinely processed, and embedded in paraffin. Sections of the paraffin-embedded tissues 5 μm thick were cut for histology. The histology slides were deparaffinized, stained with hematoxylin and eosin (H&E), coverslipped, and analyzed microscopically.

### Immunohistochemistry

Sections from paraffin-embedded blocks containing tracheobronchial lymph node (aerosol study only), inguinal lymph node, liver and spleen were sectioned, deparaffinized, rehydrated, subjected to methanol-hydrogen peroxide block, and rinsed, and antigen retrieval was performed by incubation with TRIS/EDTA Buffer at pH 9.0 for 30 min at 97°C. An immunoperoxidase assay system was used according to the manufacturer's recommendations (Envision System; DAKO lnc., Carpinteria, CA). A serum-free protein block (Life Technologies, Grand Island, NY) plus 5% normal goat serum was applied for 30 minutes followed by staining with a MARV/Angola mouse monoclonal antibody (at a dilution of I: 4000 in 10% normal goat serum) at ambient temperature for 30 min. A horseradish peroxidase-conjugated anti-mouse antibody (Envision kit) was added, and the sections were incubated for 30 min at ambient temperature. All sections were exposed to DAB permanent chromogen (DAKO, Carpinteria, CA) for 5 min, rinsed, counter-stained with hematoxylin, dehydrated, and coverslipped with Permount (Thermo Fisher Scientific, Waltham, MA). The intensity of staining for antigen was semi-quantitatively documented using the following scale: + for 1–25 reactive cells/high power field (hpf); ++ for 25–50 reactive cells/hpf; +++ for > 50 reactive cells/hpf.

## Supporting Information

S1 Fig Supplemental hematology. Hematology was performed following each blood collection. (A), (C), and (E) show hematology parameters for IM-exposed dose groups. (B), (D), and (F) show hematology parameters for aerosol-exposed dose groups.
(PDF)

S2 Fig Supplemental clinical chemistries. Clinical chemistry measurements were obtained following each blood collection. (A), (C), and (E) show chemistry parameters for IM-exposed dose groups. (B), (D), and (F) show chemistry parameters for aerosol-exposed dose groups.
(PDF)
