# Supplementary material for: Dose Response of MARV/Angola Infection in Cynomolgus Macaques following IM or Aerosol Exposure
Source: PLoS One. 2015 Sep 28;10(9):e0138843. doi: 10.1371/journal.pone.0138843 (PMC4586374; doi:10.1371/journal.pone.0138843)
Supplement: S1 Fig — Hematology was performed following each blood collection. (A), (C), and (E) show hematology parameters for IM-exposed dose groups. (B), (D), and (F) show hematology parameters for aerosol-exposed dose groups. (PDF) [file pone.0138843.s001.pdf]

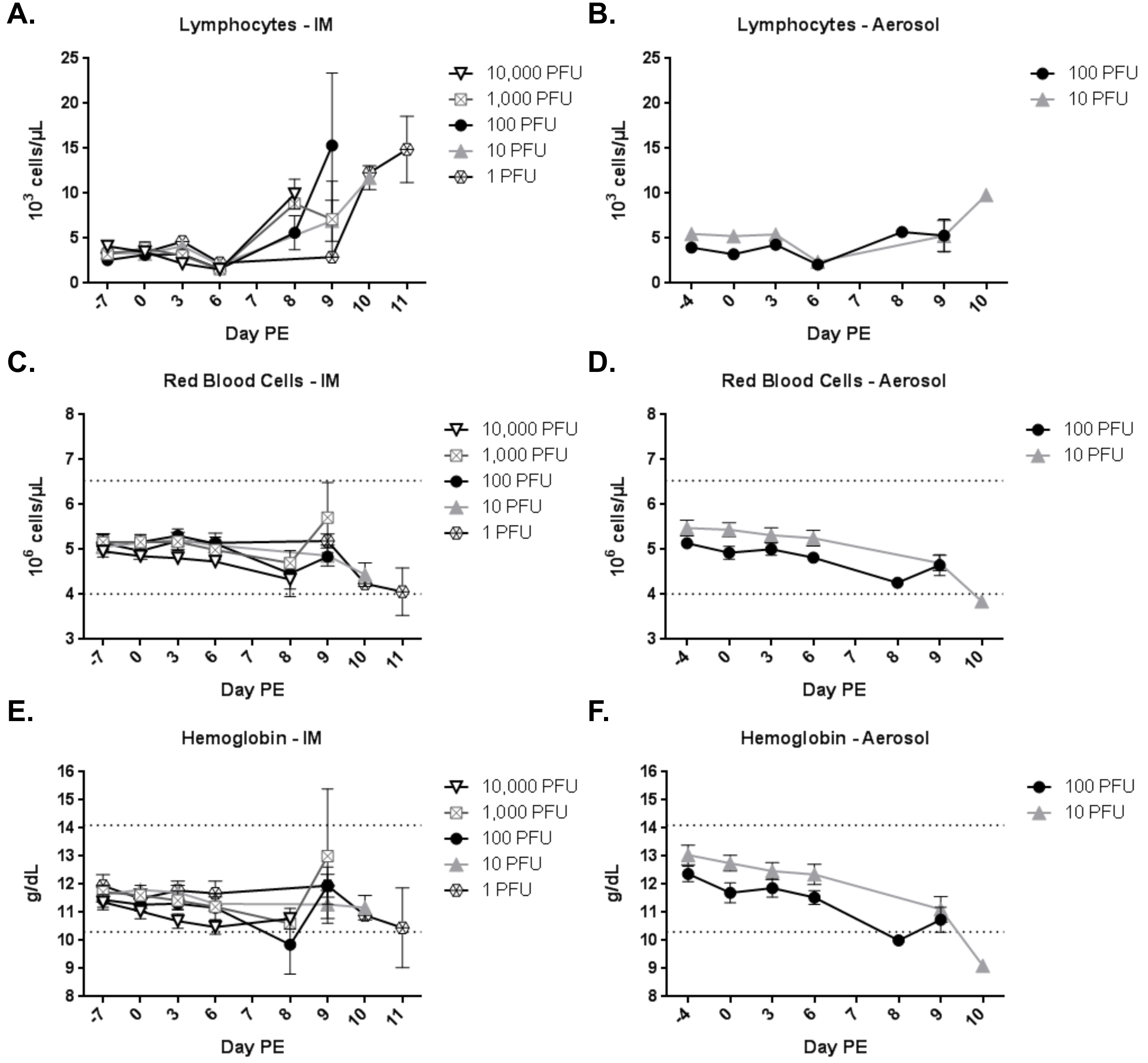
A.
Lymphocytes - IM
10^3 cells/μL
Day PE
10,000 PFU
1,000 PFU
100 PFU
10 PFU
1 PFU
B.
Lymphocytes - Aerosol
10^3 cells/μL
Day PE
100 PFU
10 PFU
C.
Red Blood Cells - IM
10^6 cells/μL
Day PE
10,000 PFU
1,000 PFU
100 PFU
10 PFU
1 PFU
D.
Red Blood Cells - Aerosol
10^6 cells/μL
Day PE
100 PFU
10 PFU
E.
Hemoglobin - IM
g/dL
Day PE
10,000 PFU
1,000 PFU
100 PFU
10 PFU
1 PFU
F.
Hemoglobin - Aerosol
g/dL
Day PE
100 PFU
10 PFU
