# Supplementary material for: Dose Response of MARV/Angola Infection in Cynomolgus Macaques following IM or Aerosol Exposure
Source: PLoS One. 2015 Sep 28;10(9):e0138843. doi: 10.1371/journal.pone.0138843 (PMC4586374; doi:10.1371/journal.pone.0138843)
Supplement: S2 Fig — Clinical chemistry measurements were obtained following each blood collection. (A), (C), and (E) show chemistry parameters for IM-exposed dose groups. (B), (D), and (F) show chemistry parameters for aerosol-exposed dose groups. (PDF) [file pone.0138843.s002.pdf]

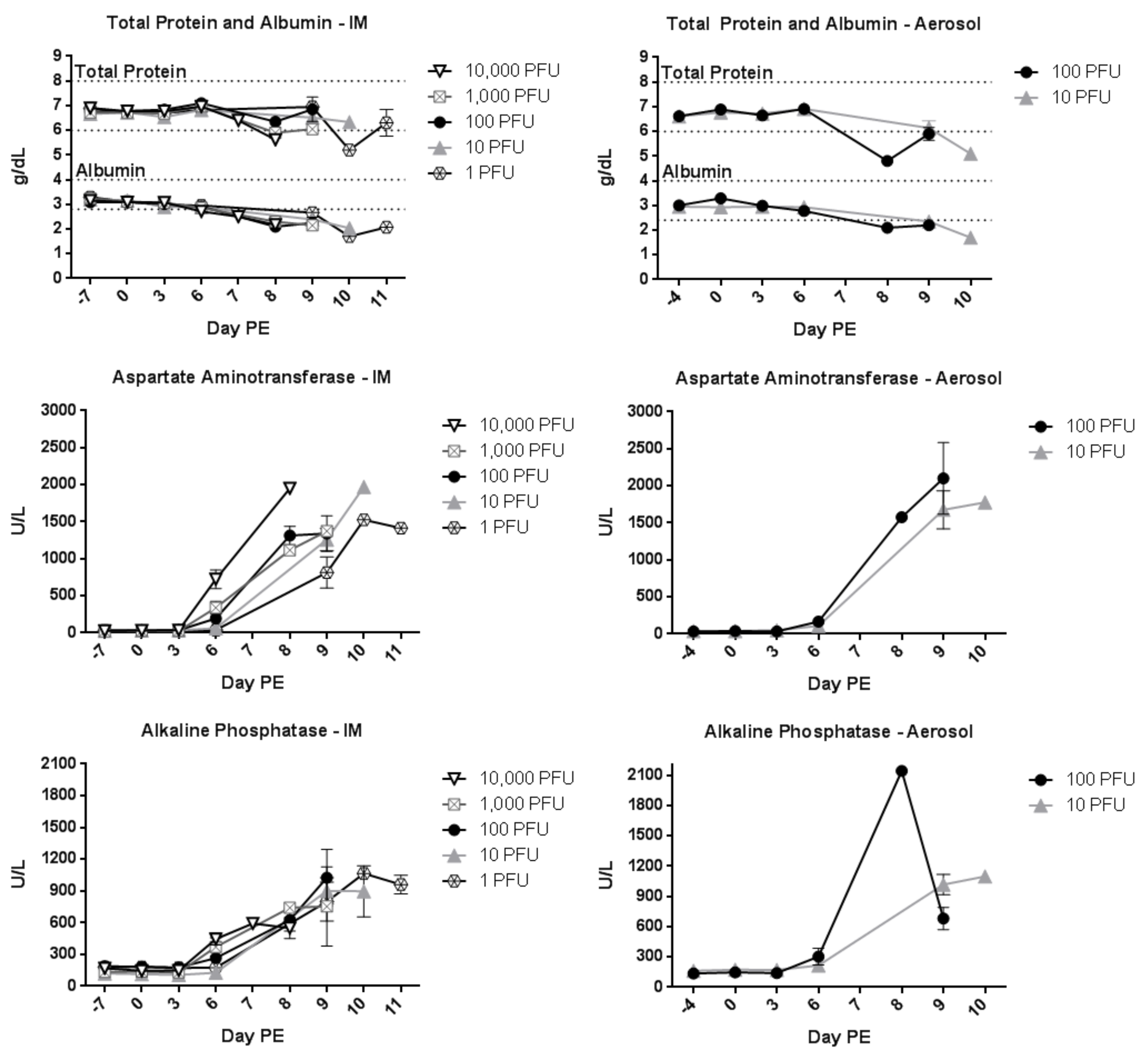
Total Protein and Albumin - IM
Total Protein
Albumin
g/dL
Day PE
10,000 PFU
1,000 PFU
100 PFU
10 PFU
1 PFU
Total Protein and Albumin - Aerosol
Total Protein
Albumin
g/dL
Day PE
100 PFU
10 PFU
Aspartate Aminotransferase - IM
U/L
Day PE
10,000 PFU
1,000 PFU
100 PFU
10 PFU
1 PFU
Aspartate Aminotransferase - Aerosol
U/L
Day PE
100 PFU
10 PFU
Alkaline Phosphatase - IM
U/L
Day PE
10,000 PFU
1,000 PFU
100 PFU
10 PFU
1 PFU
Alkaline Phosphatase - Aerosol
U/L
Day PE
100 PFU
10 PFU
